# Hospitals as Training Centres for Nurses

**Published:** 1920-12-25

**Authors:** 


### Hospitals as Training Centres for Nurses.

Miss Beatrice Monk, in an inspiring paper in this month's *London Hospital Gazette* on the future of the nursing profession, calls attention to the importance of that aspect of hospital work which centres round the nurse training-schools. It is common enough to hear prominence given to the work of medical education carried out by the hospitals, but how many people realise that the Nursing Service, on which their safety as well as comfort depends, is practically subsidised by the hospitals?

Miss Monk lays particular stress on the co-operation between the surgeon and the nursing sister. "How often does a surgeon perform a big operation on a patient, and after doing so, though his patient looks more dead than alive when he leaves the operating table, has every confidence of the patient's ultimate recovery, because he has handed the patient over to an experienced trained sister?" She believes that nurses in institutions are still grievously underpaid, and she looks for some hospital "to take the plunge and once and for all make the world recognise the value of nurses and their work." The Matron of the London Hospital contrasts the £3 a week which can now be earned by almost unskilled workers with the £100 a year, plus board and lodging, which a nurse earns after perhaps twelve or thirteen years' work at her profession, and declares that until the trained nurse is given the right status, an adequate salary for the position and knowledge she has worked for and taken years to gain, there will always be a dearth of nurses. There is just reason for the London Hospital to be proud of the fact that, in spite of all its financial difficulties, big increases of salaries are to be given to the nursing staff from January 1.

We cordially agree with Miss Monk that the splendid work which hospitals perform in training nurses for the public service ought to be kept continually before the nation in the difficult task of raising money to keep the doors open. If every member of the general public who has enjoyed the services of a "London" nurse during the last ten years would send a five-pound note this Christmas to Lord Knutsford, as a tribute to the service rendered in fitting her for her ministrations, the problems which face the treasurer would be considerably eased.

